# Evolutionary history of the p53 family DNA-binding domain: insights from an *Alvinella pompejana* homolog

**DOI:** 10.1038/s41419-022-04653-8

**Published:** 2022-03-07

**Authors:** Qiang Zhang, Dimitrios-Ilias Balourdas, Bruno Baron, Alon Senitzki, Tali E. Haran, Klas G. Wiman, Thierry Soussi, Andreas C. Joerger

**Affiliations:** 1 Department of Neuroscience, Biomedicum, Karolinska Institutet, Stockholm, Sweden; 2 Institute of Pharmaceutical Chemistry, Goethe University, Max-von-Laue-Str. 9, 60438 Frankfurt am Main, Germany; 3 Buchmann Institute for Molecular Life Sciences and Structural Genomics Consortium (SGC), Max-von-Laue-Str. 15, 60438 Frankfurt am Main, Germany; 4 Plateforme de Biophysique Moléculaire, Centre de Ressources et de Recherches Technologique (C2RT), Institut Pasteur, 75015 Paris, France; 5 Department of Biology, Technion-Israel Institute of Technology, Technion City, Haifa, 32000 Israel; 6 Department of Oncology-Pathology, Bioclinicum, Karolinska Institutet, Stockholm, Sweden; 7 Department of Immunology, Genetics and Pathology, Uppsala University, Uppsala, Sweden; 8 Sorbonne Université, UPMC Univ Paris 06, 75005 Paris, France

**Keywords:** Transcription factors, Tumour-suppressor proteins, X-ray crystallography, DNA damage response, Protein folding

## Abstract

The extremophile *Alvinella pompejana*, an annelid worm living on the edge of hydrothermal vents in the Pacific Ocean, is an excellent model system for studying factors that govern protein stability. Low intrinsic stability is a crucial factor for the susceptibility of the transcription factor p53 to inactivating mutations in human cancer. Understanding its molecular basis may facilitate the design of novel therapeutic strategies targeting mutant p53. By analyzing expressed sequence tag (EST) data, we discovered a p53 family gene in *A. pompejana*. Protein crystallography and biophysical studies showed that it has a p53/p63-like DNA-binding domain (DBD) that is more thermostable than all vertebrate p53 DBDs tested so far, but not as stable as that of human p63. We also identified features associated with its increased thermostability. In addition, the *A. pompejana* homolog shares DNA-binding properties with human p53 family DBDs, despite its evolutionary distance, consistent with a potential role in maintaining genome integrity. Through extensive structural and phylogenetic analyses, we could further trace key evolutionary events that shaped the structure, stability, and function of the p53 family DBD over time, leading to a potent but vulnerable tumor suppressor in humans.

## Introduction

The tumor suppressor p53 is an ideal paradigm for studying the effect of disease mutations and principles of protein evolution. Upon cellular stress, such as DNA damage or oxidative stress, p53 induces transcription of target genes triggering cell-cycle arrest and DNA repair, or apoptosis if the DNA damage is beyond repair [1, 2]. Besides these classical functions, p53 controls many other cellular processes, including senescence, angiogenesis, metabolism, and stemness [1, 2]. The *TP53* gene is inactivated by mutation in about half of all human cancers [1, 3, 4]. Most oncogenic p53 mutations are missense mutations in the highly conserved DNA-binding domain (DBD) [5, 6] and have been classified as either DNA-contact mutations or structural mutations [7–9]. Contact mutations remove essential DNA-contact residues [10], whereas structural mutations destabilize the DBD to various degrees, resulting in the unfolding of the mutant protein at physiological conditions, followed by rapid aggregation [11, 12]. Several strategies to reactivate mutant p53 in cancer are being explored, including small-molecule stabilizers of the thermolabile mutant Y220C [13, 14], metallochaperones for zinc-binding deficient mutants [12, 15], and cysteine-binding compounds APR-246/MQ, 2-sulfonylpyrimidines, and arsenic trioxide that target a wider range of p53 mutants [16–19]. Understanding the structure of p53, the factors that govern its stability, and how it responds to mutation is therefore essential for the development of mutant p53 rescue drugs.

**Fig. 1:**
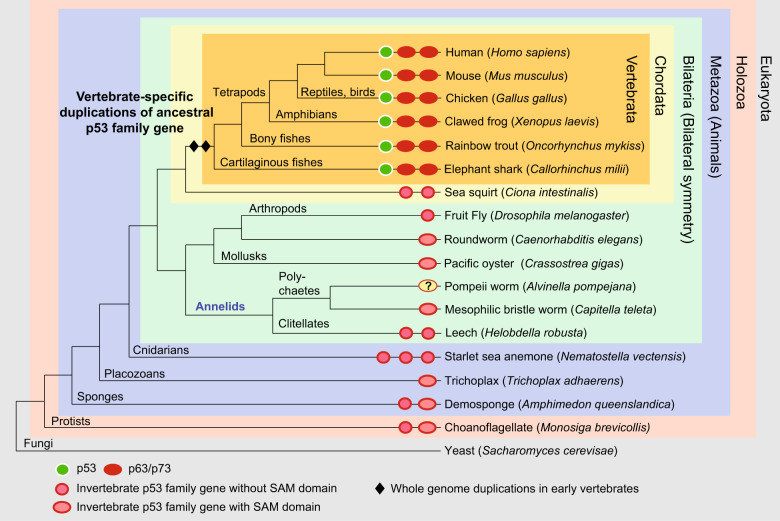
Simplified evolutionary tree of p53 family proteins. Vertebrates typically have three p53 family members, p53, p63, and p73, as a result of two gene duplications at the beginning of vertebrate evolution. Multiple copies as a result of independent gene duplications are also found in several invertebrate lineages. The invertebrate p53 family genes in this tree are classified solely based on the presence or absence of a SAM domain in the predicted proteins (Ensembl Metazoa release 52 and Ensembl Protists release 52), which is a characteristic feature of vertebrate p63 and p73 proteins, but absent in vertebrate p53. The genome of the Pompeii worm (*Alvinella pompejana*), a member of the annelid worms, has not yet been published. Comparison with the published genome of another polychaete annelid, *Capitella teleta*, suggests the presence of a single p53 family gene in this organism that contains an extended p63/p73-like tetramerization domain with a second helix [77] and a SAM domain. From the analysis of the chicken genome, only p63 and p73 proteins are predicted, but a p53 transcript has been verified experimentally [78]. Branch lengths in this tree do not reflect evolutionary distance. The figure has been adapted from Joerger and Fersht [1].

The susceptibility of p53 to inactivation by destabilizing mutations is deeply rooted in its evolutionary history. The *TP53* gene and the other two members of the family, *TP63* and *TP73*, have evolved through a complex pathway that started with the beginning of animal life more than one billion years ago from a p63/p73-like common ancestor (Fig. 1) [1, 20]. p53 family genes were identified in the ancient placozoan *Trichoplax adhaerens* and in choanoflagellates, the closest living relatives of metazoans [21, 22]. A more recent bioinformatics study suggested the presence of p53 homologs in several unicellular holozoans [23]. Then, via a series of gene duplications as well as gain and loss of functional domains, e.g., loss of the sterile alpha motif (SAM) domain in the human p53 lineage, evolution has led to the stabilization of three genes in vertebrates, resulting in three p53 family proteins with overlapping and distinct functions: p53, p63, and p73 [1, 24, 25]. The DBD is highly conserved in the three paralogs, but p53 has evolved at a much faster rate than p63 and p73 [20]. This evolution goes along with increased sensitivity of the p53 DBD to structural stress compared with the more stable paralogs p63 and p73. This was first revealed in studies showing that the *Xenopus* p53 protein behaves like a temperature-sensitive human p53 cancer mutant [26, 27]. Subsequent studies revealed that the thermostability of the p53 DBD correlates with the organismal temperature of endothermic animals and temperatures for optimal development of ectothermic vertebrates [28–30].

It is worth pointing out that lower intrinsic thermodynamic stability of a protein domain correlates with more efficient degradation by the ubiquitin 26S proteasome system [31]. p53 has a very short half-live in human cells and is tightly regulated via a feedback loop in which p53 induces the transcription of the E3 ubiquitin ligase MDM2, which (in conjunction with the paralog MDMX) marks p53 for proteasomal degradation [32, 33]. This negative feedback loop, in combination with low intrinsic conformational stability, ensures that p53 is only active as long as needed, preventing p53-induced cell death of healthy cells. Conformational stability and plasticity of the p53 DBD are therefore key structural properties that determine both regulation and function of p53.

To gain further insights into the evolutionary history of p53 and factors that govern the stability of p53 family proteins, we have characterized the DBD of a p53 homolog from the extremophilic annelid *Alvinella pompejana*, which lives in tubes at the edge of hydrothermal vents at the bottom of the Pacific. It is one of the most extremophilic species on the planet, tolerating temperatures of up to 80 °C, high pressure, and high concentrations of heavy metals and hydrogen sulfides [34, 35]. We show that it has a p53/p63-like DBD that is more thermostable than all vertebrate p53 DBDs tested so far, but, intriguingly, not as stable as human p63. Through extensive structural and phylogenetic analyses of vertebrate and invertebrate p53 family proteins, we provide unique insights into the structural evolution of the p53 family and trace key evolutionary events that shaped the structure and function of its DBD.

## Results and discussion

### Phylogenetic and structural analysis of the *A. pompejana* p53 homolog

We performed a BLAST search of the *A. pompejana* expressed sequence tag (EST) database of the Max Planck Institute for Developmental Biology, Tübingen, Germany [36] to search for p53 family proteins and obtained a hit for a transcript containing a p53/p63-like DBD (N72937). Searching the NCBI EST database yielded two further transcripts from the posterior end of *A. pompejana* (GenBank ID GO114002.1 and GO114003.1) covering the same sequence range (Supplementary Fig. S1). The genome of *A. pompejana* is not yet available, but the genomes of two other annelids, the marine polychaete *Capitella teleta* and the freshwater leech *Helobdella robusta*, have been released [37]. A BLAST search of these genomes in the Ensembl genome browser revealed a single p53 family gene in both species. In the case of *C. teleta*, the putative p53 family gene has a p63/p73-like domain architecture featuring an N-terminal transactivation domain, a DBD, an extended tetramerization domain with its characteristic second helix [38], and a SAM domain (Uniprot entry R7UHV7). The same scenario was also found for evolutionarily related mollusks (Fig. 1). Intriguingly, the *C. teleta* homolog even shows conservation of the β-strand regions in the N-terminal transactivation domain and the C-terminal inhibitory domain region of human p63 (TAp63α); these regions are predicted to assemble into a single β-sheet in human p63 overexpressed in unstressed oocytes, resulting in the formation of inactive dimers in the absence of DNA damage [39, 40].

The two p53 homologs predicted from the *H. robusta* genome lack the C-terminal SAM domain, hinting at a potential deletion of this domain in the clitellate lineage after the divergence of polychaetes and clitellates. However, other structural elements are also missing in these predicted transcripts: one lacks the transactivation domain and the N-terminal segment of the DBD (Uniprot entry T1EZJ4), reminiscent of the Δ133 isoform of human p53 [41], while the other has the full DBD (with a key DNA-contact residue mutated) but no intact tetramerization domain (UniProt entry T1EE77).

We also performed a BLAST search of the *A. pompejana* ESTs for an MDM2/4-like protein to gain insights into a potential degradation pathway via the ubiquitin/proteasome system, but no hits were found. For the *C. teleta* genome, however, we retrieved hits for the key functional domains of MDM2, consistent with an earlier report [42] on the presence of an MDM-like gene in this annelid including all four functional domains (p53-binding region, acidic domain, C4 zinc finger, and C-terminal RING domain).

**Fig. 2:**
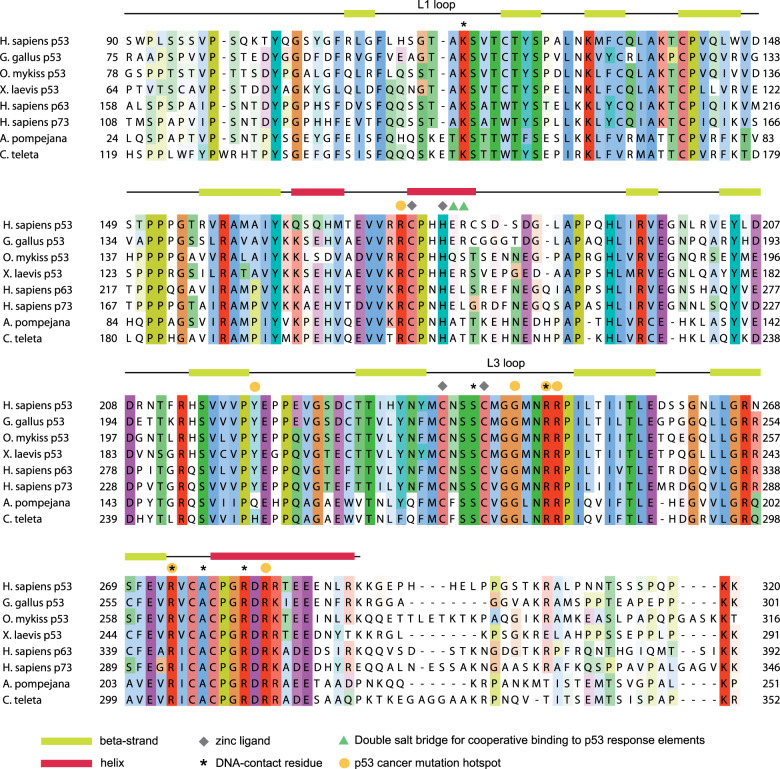
Sequence alignment of p53 family DNA-binding domains. The partial sequence of the *Alvinella pompejana* p53 homolog, including the full DBD, was aligned with the sequences of human p53, p63, and p73, and the p53 homolog from *Capitella teleta* using MUSCLE [68] and visualized with Jalview [69]. Amino acid residues are colored according to the Clustal X color scheme based on sequence conservation and similarity. The secondary-structure assignment above the alignment refers to the structure of the human p53 DBD (2XWR) [79]. Strictly conserved DNA-contact and zinc-binding residues as well as p53 cancer mutation hotspot sites are highlighted. UniProt accession codes: human p53, P04637; human p63, Q9H3D4; human p73, O15350; chicken p53 (*Gallus gallus*), P10360; rainbow trout p53 (*Oncorhynchus mykiss*), P25035; *Xenopus laevis* p53, Q7T1D0; *C. teleta* p53 homolog, R7UHV7. The numbering of the *A. pompejana* p53 homolog is based on EST N72937.

The sequence of the *A. pompejana* DBD is highly conserved (Fig. 2), showing 47% sequence identity with the human p53 DBD. The conservation with p63 and p73 is even higher (58% and 56% identical residues, respectively), consistent with the notion that the ancestral protein of metazoan p53 proteins was more p63-like [1, 23]. The pairwise sequence identity between the two polychaete worm DBDs is above 80%.

**Fig. 3:**
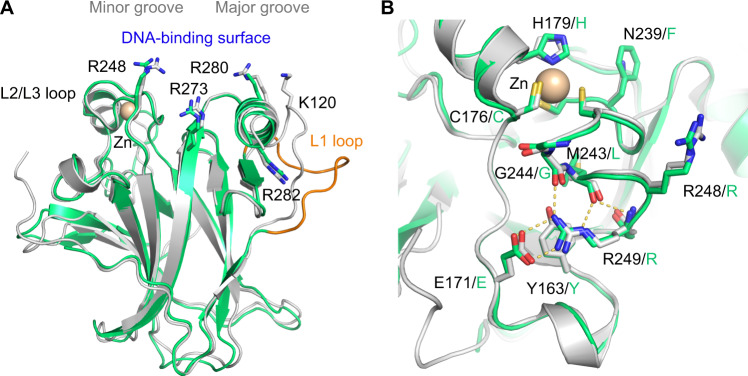
Crystal structure of the *Alvinella pompejana* p53 DBD. **A** Cartoon representation of the overall structure of the *A. pompejana* DBD (green; chain A) superimposed onto the structure of human p53 DBD in its unbound state (gray; PDB code 2XWR) [79]. Residue numbers given refer to the human protein. **B** Conservation of zinc coordination and the Arg249-mediated polar interaction network in the L3 loop region of the *A. pompejana* DBD. Stabilization of the hairpin conformation of the L3 loop is key for positioning the DNA contact residue Arg248 for docking to the minor groove of DNA response elements. The oncogenic R249S mutation destabilizes the human protein and impairs DNA binding [80]. The structure of the *A. pompejana* DBD is shown in green superimposed onto the structure of human p53 DBD in gray.

We determined the crystal structure of the *A. pompejana* DBD at 1.92 Å resolution (Fig. 3 and Supplementary Table S1). The overall structure consists of an immunoglobulin-like β-sandwich, which serves as a scaffold for an extended DNA-binding surface. The latter is formed by a loop-sheet-helix motif and two large loops that are held together by zinc coordination. Residue numbering in the following refers to the position in the human p53 protein, unless otherwise stated, because the sequence of the full-length *A. pompejana* p53 homolog is not known yet. The zinc-binding site (Cys176, His179, Cys238, and Cys242) is conserved in *A. pompejana*. Key DNA-contact residues are also conserved: Arg248 in Loop L3 for minor groove binding, Arg273, which interacts with the phosphate backbone, and Arg280 in the C-terminal helix that interacts with guanine in the major groove in human p53-DNA complexes. Residues important for the structural integrity of the DNA-binding surface and for positioning DNA-contact residues are conserved as well, e.g., Arg175 or Arg249, which stabilizes the conformation of the L3 loop orienting Arg248 towards minor groove binding (Fig. 3B).

Structural differences were found mainly on the surface and in loop regions, for example in the L1 loop, which plays a unique role in DNA recognition by human p53. Depending on the position of the DBD within the tetrameric p53–DNA complex and the particular sequence of the target site, this loop makes either direct contact with DNA via Lys120 or adopts a recessed conformation without direct DNA contact [43, 44]. Moreover, acetylation of Lys120 modulates human p53 target gene specificity [45]. In the p53 homologs from the two model organisms *Drosophila melanogaster* and *Caenorhabditis elegans*, the L1 loop has significantly diverged and is much shorter [46, 47], indicating that it does not play the same role in DNA binding as in the human protein. In *A. pompejana*, there is much less divergence in this loop, apart from a one-residue insertion at its N-terminal base. However, the overall conformation differs significantly from that seen in human p53, adopting a much more recessed orientation (Fig. 3A), with a high degree of disorder observed in most chains of the asymmetric unit. The higher structural conservation of the DBD in *A. pompejana* is consistent with the observation that *A. pompejana* genes generally display a slow evolutionary rate compared with the fast-evolving *C. elegans* and *Drosophila* genomes [48], despite adaptation to a challenging habitat.

### Insights into the evolution of p53 DNA-binding cooperativity

An interesting structural feature of human p53 is a double salt bridge between two DBDs via Glu180 and Arg181 that is crucial for the cooperative binding of a p53 tetramer to its response elements [44, 49]. We found that this double salt bridge is a typical feature of vertebrate p53 sequences, including cartilaginous fishes, with rainbow trout p53 being a notable exception (Fig. 2). One of those salt-bridge partners, Arg181, is replaced by leucine in p63 and p73, which, accordingly, do not show binding cooperativity [50]. In *A. pompejana*, both charged residues are replaced by an alanine and threonine, respectively, again highlighting the more p63/p73-like nature of the DBD in this organism. The comparison with sequences of other invertebrate p53 homologs where this p53 signature motif is also missing (including the two paralogs in the tunicate *Ciona intestinalis*) and its absence in lamprey p53 suggest that this double salt bridge motif did not pre-exist in the ancestral protein of the vertebrate p53 family but has specifically evolved in the p53 lineage with the emergence of cartilaginous fishes.

### Thermostability of the *A. pompejana* DBD

**Fig. 4:**
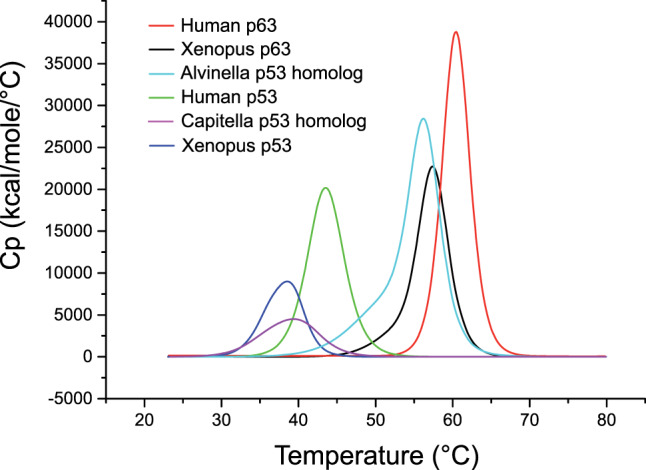
Thermostability of different p53 family DBDs. Raw data of the differential scanning calorimetry (DSC) analysis for the DBDs of *A. pompejana* p53 homolog, *C. teleta* p53 homolog, human p63, *Xenopus* *laevis* p63, human p53, and *Xenopus laevis* p53, recorded at a heating rate of 300 °C/h. *T*_m_ values measured at a much lower heating rate of 60 °C/h were on average about 2–3 °C lower with both DSC and DSF assays. It has been shown previously that p53 family proteins denature irreversibly and that the measured apparent *T*_m_ depends on the heating rate but approximates the true *T*_m_ if the heating rate is fast enough [29, 81].

We determined the apparent melting temperature (*T*_m_) of the *A. pompejana* DBD and selected vertebrate and invertebrate p53 family DBDs by differential scanning calorimetry (DSC). In most cases, those stability measurements were complemented by differential scanning fluorimetry (DSF) using the Prometheus system, which monitors intrinsic tryptophan or tyrosine fluorescence, and by circular dichroism (CD) (Fig. 4 and Table 1). There was a very good agreement between the results obtained with the three methods, and the same trend was also observed by conventional DSF with the fluorescent dye SYPRO orange (Supplementary Fig. S2). The *T*_m_ values in the following refer to the DSC data.

**Table 1:** Apparent melting temperatures of p53 family DBD variants.

DBD variant	*T*_m_ (°C)^a^ DSC	*T*_m_ (°C) Prometheus	*T*_m_ (°C) CD
*A. pompejana* p53 homolog	56.2	56.9	53.5
*C. teleta* p53 homolog	39.1	41.3	–
Human p63	60.4	60.0	62.8
*Xenopus* p63	57.5	57.5	55.7
Human p53	43.6	41.3	41.3
*Xenopus* p53	38.2	38.3	36.3
Chicken p53	54.0	–	–
Rainbow trout p53	37.5	–	–
Human p53 mutant R175H	36.1	–	–

^a^All stability measurements were performed at a heating rate of 300 °C/h, except for chicken and rainbow trout p53 and the R175H cancer mutant, which were measured at a heating rate of 200 °C/h.

As expected, the *A. pompejana* DBD had a very high thermostability, with a *T*_m_ of 56 °C. The DBD of a homolog from the mesophilic annelid *C. teleta*, which is commonly found in shallow and brackish waters on the east and west coast of North America, was significantly less stable by up to 17 °C across the different stability measurement techniques. The human p63 DBD had the highest thermostability of all variants tested (*T*_m_ = 60 °C), whereas the human p53 DBD had a *T*_m_ of only 44 °C. Consistent with published data [28, 29], the thermodynamic stability of other vertebrate p53 DBDs correlated with differences in organismal temperature or living conditions in the case of cold-blooded species. The p53 DBD of chicken, which have a higher body temperature (40–44 °C) than humans [28], was significantly more stable than the human p53 DBD, and the DBD of the cold-blooded clawed frog *Xenopus laevis* was significantly less stable. The stability of the p53 DBD from another cold-blooded animal, rainbow trout, which is native to cold-water tributaries of the North Pacific, was even slightly lower (*T*_m_ = 37.5 °C) than that of the *Xenopus* p53 DBD, further supporting the idea that vertebrate p53 DBDs evolved to be intrinsically unstable [28, 29]. Low intrinsic stability of p53 may therefore be of functional importance, for example facilitating the rapid cycling between folded and unfolded states, thereby increasing p53 turnover and resulting in a more stringent regulation of cellular p53 activity. The *Xenopus* p63 DBD, however, exhibited a very high thermostability (*T*_m_ = 58 °C), which was expected, given the high sequence conservation between human and *Xenopus* p63 (only three variations in the DBD). The DBDs of human and *Xenopus* p53 share only 67% sequence identity, highlighting the higher divergence of vertebrate p53 compared with p63 and p73, which have retained more ancestral features.

### Structural basis for increased stability of the *A. pompejana* DBD and evolutionary history of its hydrophobic core packing pattern

**Fig. 5:**
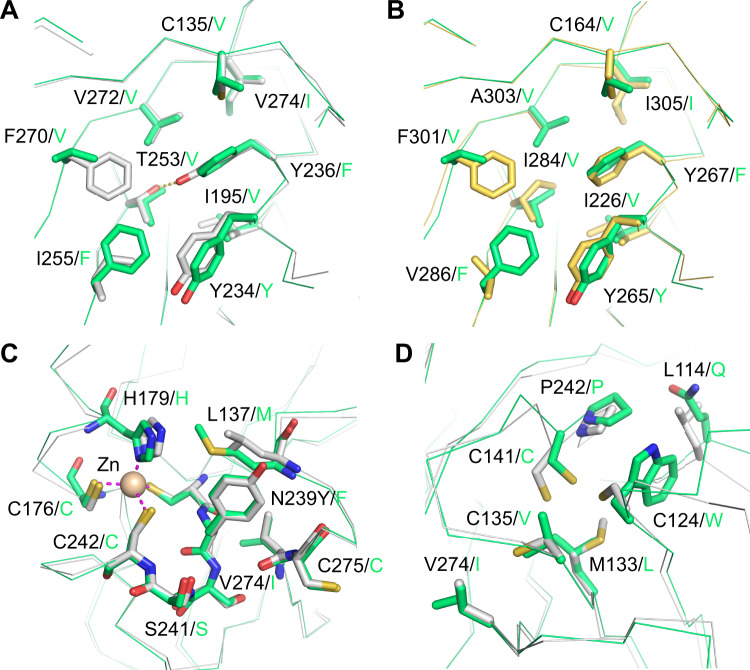
Stabilizing interactions in the DBD of the *Alvinella pompejana* p53 homolog. **A**, **B** Hydrophobic core of the *A. pompejana* DBD. Superposition of the *A. pompejana* DBD (green) onto the structure of human p53 DBD (gray; PDB code 2XWR) [79] in (**A**) and the structure of human p63 DBD (PDB code 3QYN; yellow) [82] in (**B**) shows key differences in the hydrophobic core of the *A. pompejana* protein. Most notably, a phenylalanine and a neighboring smaller hydrophobic amino acid have swapped places when comparing the *A. pompejana* and the human DBDs (residues 255 and 270 in human p53). A non-saturated hydrogen bond found in the hydrophobic core of the human protein (orange dotted line) is replaced by equivalent hydrophobic residues seen in the human p63/p73 structure. **C** Superposition of the *A. pompejana* p53 DBD onto the structure of the superstable quadruple mutant M133L/V203A/N239Y/N268D of human p53 (PDB code 1UOL; gray) [55] shows a phenylalanine in the *A. pompejana* structure at the position of the stabilizing N239Y substitution in human p53 next to the zinc-binding site. **D** The same superposition as in panel C but focusing on the location of the cysteine cluster in the human protein shows that this cysteine cluster is absent in the *A. pompejana* DBD. Cys124 in the human p53 DBD is substituted by tryptophan in the *A. pompejana* DBD.

**Fig. 6:**
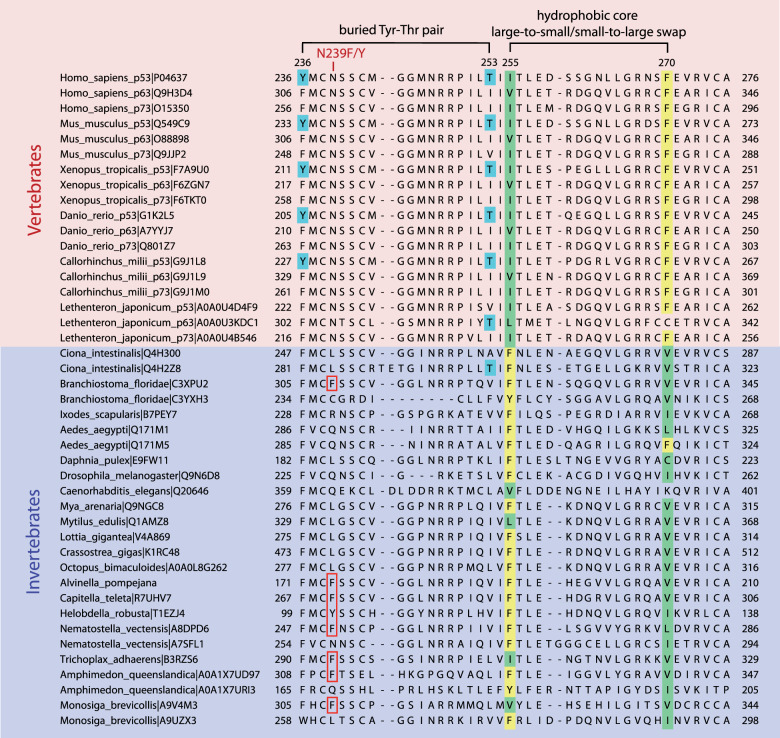
Reshaping of the hydrophobic core of p53 family proteins during evolution. A sequence alignment of vertebrate and invertebrate p53 family DBDs shows a switch of the packing pattern of the DBD hydrophobic core via residues 255 and 270. The aromatic swap illustrated in Fig. 5A appears to have occurred at the transition from invertebrates to vertebrates. Also highlighted is an unsaturated hydrogen-bond pair in the hydrophobic core of vertebrate p53 DBDs (residues 236 and 253, human p53 numbering). UniProt accession numbers are given after the name of each species. The numbering of the *A. pompejana* p53 homolog is based on the translation of EST N72937.

The high thermostability of the *A. pompejana* DBD can be explained by the high sequence conservation with the more stable human p63 DBD, which is generally characterized by an optimized packing of its hydrophobic core [50]. Inefficient core packing has been attributed to the low stability of p53 DBDs from cold-blooded vertebrates [28]. The effect of inefficient packing is demonstrated by the cancer-associated cavity-creating F270L mutation in the hydrophobic core of the human p53 DBD. This mutation reduces the thermodynamic stability of the DBD by 4 kcal/mol, resulting in unfolding at a physiological temperature [8, 51]. Intriguingly, *A. pompejana* has a similar large-to-small substitution at this position, F270V, but in this case, the crystal structure of the DBD shows that the substitution is compensated for by a small-to-large substitution of a neighboring residue, I255F, which maintains optimal packing of the hydrophobic core (Fig. 5A, B). Again, as with F270L/V, the I255F mutation on its own is highly destabilizing in human p53 and hence oncogenic (Supplementary Fig. S3) [8]. This inverted packing pattern is found in most invertebrate p53 family sequences, from choanoflagellates up to tunicates (Fig. 6), whereas all vertebrate p53 family proteins, including the jawless fish lamprey, have the 255/270 packing pattern found in human p53 (with a distinct variation of the smaller residue in p63). This observation suggests that the mutations leading to this particular repacking of the DBD core, possibly via a metastable intermediate, occurred shortly before the emergence of vertebrates during the Cambrian explosion.

Simultaneous mutation of Y236F and T253I stabilizes human p53 by removing a non-saturated hydrogen bond from the hydrophobic core of the protein [52, 53]. This variation is found in p63/p73, and a comparable hydrophobic pattern is also found in the *A. pompejana* homolog (Fig. 5A, B), which has retained p63/p73-like stabilizing features that were most likely already present in the ancestral protein. Again, an indication that p53 has evolved to be only marginally stable. There is an interesting variation close to the zinc-binding site that may contribute to protein stability that is not seen in the p63/p73 homologs. The second-site suppressor mutation N239Y of human p53 stabilizes the DBD by about 1.2 kcal/mol and has been used to generate a stabilized p53 variant for biophysical studies [54, 55]. Both *A. pompejana* and *C. teleta* also have an aromatic residue, phenylalanine, at this position, which forms similar stabilizing packing interactions as the tyrosine in the stabilized human p53 variant (Figs. 3 and 5C). *H. robusta* actually has a tyrosine at this position. Other than in annelids, we found a tyrosine or phenylalanine at this position almost exclusively in p53 family genes of species further down the evolutionary tree, including sea anemones, sponges, and unicellular holozoans (cf. Fig 1, Fig. 6 and sequences of putative p53 family homologs in ref. [23]).

The human p53 DBD features a cluster of three cysteines (Cys124, Cys135, and Cys141) at the interface between the β-sandwich and the loop–sheet–helix motif. This region is highly dynamic in the human protein [56], and a recent study has shown that several structural p53 cancer mutants are reactivated by arsenic trioxide through coordination of these three cysteines, which compensates for the mutation-induced stability loss [19]. Cys124, which is also modified by mutant p53-targeting agents such as PRIMA-1 and APR-246 [56, 57], is replaced by a tryptophan in vertebrate p63/p73 and most invertebrate DBDs, including *A. pompejana* (Figs. 2 and 5D). The additional tryptophan-mediated hydrophobic packing interactions are likely to contribute to the increased thermostability of the *A. pompejana* DBD, and also that of the p63/p73 DBD, which is supported by mutagenesis studies on human p53 [28]. Our systematic phylogenetic analysis (Supplementary Fig. S4) suggests a gradual appearance of the three cysteines during the evolution of the p53 family, with Cys141 potentially already present in the last common ancestor of all extant animals, Cys135 appearing shortly before the radiation of vertebrates, and Cys124 last, as a vertebrate p53 specific variation first appearing in the p53 lineage of bony fishes.

Holder et al. [36] suggested that the best indicator of thermoadaptation in *A. pompejana* proteins in general is the difference in frequency of charged versus polar residues (CvP-bias) compared with mesophilic organisms. Such a trend is however not apparent when comparing the p53 family DBDs of *A. pompejana* and *C. teleta* (Supplementary Fig. S5).

### Conservation of DNA-binding properties between *A. pompejana* and human p53-family proteins

**Fig. 7:**
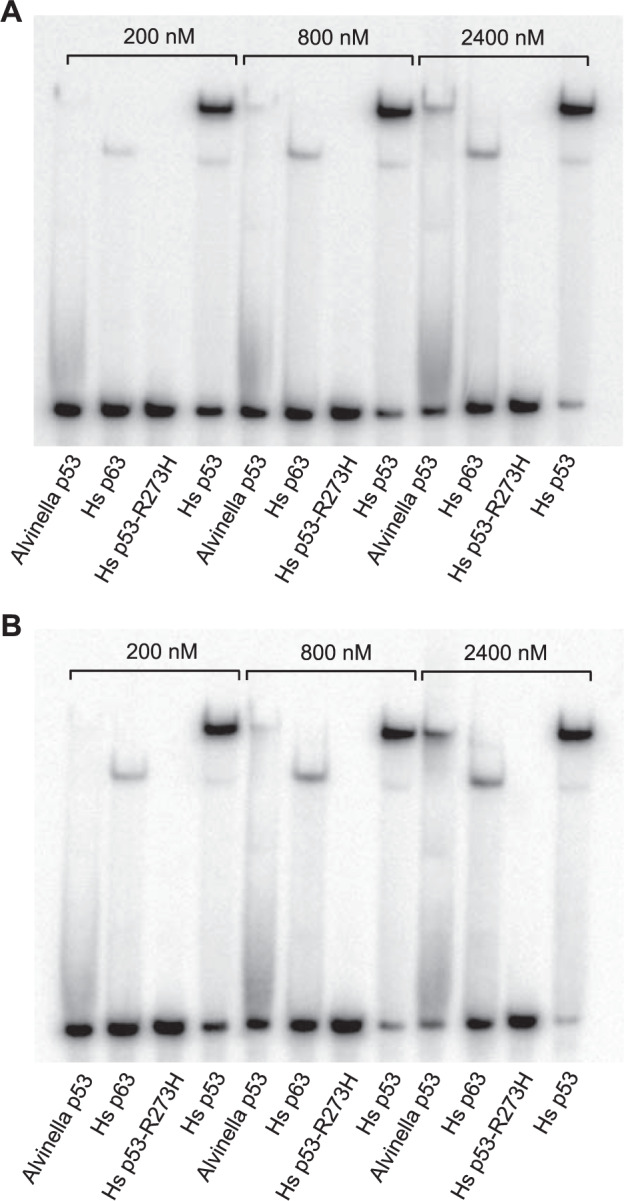
Conservation of DNA-binding properties between *Alvinella pompejana* and human p53 family DBDs, measured by electrophoretic mobility shift assay. p53 family DBDs were incubated with Con1 DNA for 1 h at 4 °C (**A**) and 20 °C (**B**) at a nominal protein concentration of 200, 800, and 2400 nM, and then run on a 6% polyacrylamide gel (37.5:1 acrylamide: bisacrylamide ratio) at the same temperature as the incubation. The DNA-binding deficient p53 cancer mutant R273H was used as a negative control.

We measured binding of the *A. pompejana* DBD to the p53 binding sequence Con1 by electrophoretic mobility shift assay (EMSA) (Fig. 7 and Supplementary Fig. S6). Con1 is a p53/p63 consensus sequence and consists of two contiguous symmetrical decameric half sites (GGGCATGTCC) [58–60]. The human p53 DBD binds to this recognition site cooperatively as a tetramer (dimer of dimers) [58]. The *A. pompejana* DBD showed the same band-shift pattern upon binding as the human p53 DBD, but the binding was only observed at higher DBD concentrations, suggesting that the *A. pompejana* DBD also binds to Con1 as a tetramer but has a lower affinity for Con1 than the human protein. Both human p63 (Fig. 7) and *Xenopus* p63 (Supplementary Fig. S6) showed a significantly faster migration than the other DBDs tested upon binding, due to migration as dimers instead of tetramers. This can be concluded from the comparable migration pattern of the bands of human p53 DBD dimers (minor band below the main tetramer band in Fig. 7) and that of human p63 DBD. The identity of the bands of p53 DBD on EMSA gels was previously established [58]. A comparison of the EMSA patterns carried out after incubation of the complexes at different temperatures showed that the p63–DNA complex appears to be the most temperature-stable as it was the least sensitive to temperature in the range from 4 to 42 °C (Fig. 7 and Supplementary Fig. S6).

Taken together, our qualitative DNA-binding studies on isolated DBDs show conservation of basic DNA-binding properties between the *A. pompejana* homolog and human p53 family proteins, despite the evolutionary distance between the two species.

### Conclusions and biological implications

Our structural and phylogenetic analyses show that the p53 homolog found in *A. pompejana* is generally more p63/p73-like and hint at mutational events during the evolution of the p53 family that reshaped the hydrophobic core of the DBD and modulated its target specificity. We were further able to show that the *A. pompejana* DBD has a much higher thermostability than the DBD of most vertebrate p53 variants, but is not as stable as that of human p63, which may be surprising at first glance. It is interesting to speculate whether the *A. pompejana* p53 homolog has evolved to be stable to adapt to the harsh environmental conditions at the edge of hydrothermal vents or whether it was already sufficiently stable to function at high temperatures. The fact that invertebrate p53 homologs are generally more p63/p73-like suggests that the ancestral protein also exhibited a high thermostability and that vertebrate p53 proteins have evolved to be intrinsically unstable rather than the other way around. Low conformational stability of vertebrate p53 has likely evolved to allow for rapid cycling between folded and unfolded state, ensuring a short half-life of transcriptionally active p53 protein in the cell. This low conformational stability, however, came at a cost, making human p53 particularly vulnerable to inactivation by destabilizing mutations.

Intriguingly, a comparative proteomics study suggests that the ancestor of all living annelid species, including those living in colder habitats, was thermophilic [61]. There is also some uncertainty about the exact temperatures *A. pompejana* is exposed to. While the posterior end of *A. pompejana* is thought to be exposed to temperatures of up to 80 °C, the anterior end is exposed to much more moderate temperatures, potentially creating a temperature gradient of up to 60 °C across the body length [34]. In addition, there is a symbiotic relationship with bacteria that cover its dorsal surface and may form an insulating layer [35]. An in vivo analysis of the heat tolerance of *A. pompejana* has shown that prolonged exposure to 50–55 °C is lethal, triggering a heat stress response [62], consistent with in vitro data on a number of *A. pompejana* proteins showing that they have melting temperatures around 45–50 °C [36, 62]. Some of the p53 homolog EST data retrieved stemmed from the posterior end of *A. pompejana*, indicating expression of the p53 family gene in the part of the worm that is exposed to the most extreme temperatures. The required thermostability of the p53/p63-like protein also strongly depends on when it is expressed during the life cycle of the organism. A potential role in germline protection as in the starlet sea anemone, where gametes but not adult cells are killed upon radiation-induced DNA damage [63], would require expression in the larval stage. Interestingly, embryonic *A. pomejana* do not tolerate extreme temperatures and can arrest their development in the larval stage when they float in colder water to colonize new vents [64].

An important question regarding the functional regulation of the *A. pompejana* p53 homolog—besides the thermodynamic and kinetic stability of its DBD—is whether its cellular protein levels are also controlled via an MDM2-like protein, or via alternative degradation pathways. We did not detect an MDM2-like transcript in the available EST data of *A. pompejana*, although an MDM2 homolog was detected in the genome of the mesophilic annelid *C. teleta* [42] and may therefore also be found in *A. pompejana*. So far, the evolutionary tree of MDM2-like E3 ligases in invertebrates is somewhat incomplete. Single MDM-family genes were identified in many, but not all, invertebrates, including placozoans, sea anemones, mollusks, and tunicates [1, 42, 65]. In addition, functional analysis of p53 pathway components of the ancient metazoan *T. adhaerens* showed that the placozoan MDM protein interacts with the p53 homolog and triggers its proteasomal degradation [21]. A gene duplication shortly before the radiation of vertebrates then led to two distinct MDM-family proteins being present in all vertebrates: MDM2 and MDMX, with the latter lacking E3 ligase activity [24, 66]. Despite some gaps in the phylogenetic tree and the complicating matter of low sequence coverage of some genomes, the overall picture that emerges is that the p53-MDM2 regulatory axis can be traced back to early metazoans and has since then tightly co-evolved, or disappeared in distinct lineages including *C. elegans* and *D. melanogaster* [42, 65].

Sequencing of the *A. pompejana* genome will reveal if it contains a single, p63-like gene with a SAM domain and autoregulatory features as found in the mesophilic annelid *C. teleta*. It will also shed more light on a potential degradation pathway involving an MDM2-like protein. Such genomic data could then form the basis for more in-depth functional studies to further elucidate the biological role of the p53 family gene in this enigmatic organism at the abyss of the ocean.

## Materials and methods

### Phylogenetic analyses

Sequences of p53 family genes/proteins in different species were retrieved from UniProt (http://www.uniprot.org/) and by a BLAST search [67] of the Ensembl genome browser (Ensembl release 102, Ensembl Metazoa release 52 and Ensembl Protists release 52, http://www.ensembl.org/index.html) and EST databases. Multiple sequence alignments were performed using MUSCLE [68] and visualized with JALVIEW [69].

### Cloning, protein expression, and purification

cDNA sequences encoding the DBD of the various p53 family proteins were cloned either in pNIC-Bsa4, a derivative of pET28a (human, *Xenopus*, chicken, and rainbow trout p53) or pET11a (human and *Xenopus* p63, *A. pompejana* and *C. teleta*). In both vectors, the DBD was fused with an N-terminal 6xHis tag followed by a TEV cleavage site. The following domain boundaries were used for cloning the DBDs (database references are given in parentheses): Human p53: S94-K292 (UniProt P04637); human p63: A164-Q362 (Uniprot Q9H3D4); *X. laevis* p53: V71-E277 (UniProt P07193); *X. laevis* p63: A70-Q268 (UniProt Q98SW0); *Oncorhynchus mykiss* p53: V85-A299 (UniProt P25035); *Gallus gallus* p53: V82-A288 (UniProt P10360); *A. pompejana* p53 homolog: T30-R231 (EST N72937) see Supplementary Fig. S1; *C. teleta* p53 homolog: P127-E325 (UniProt R7UHV7).

All expression vectors were transformed into the BL21(DE3) R3 pRARE2 phage resistant *Escherichia coli* expression strain. The expression cultures (3000 ml) were incubated at 37 °C in terrific broth supplemented with 8 g/l glycerol, 0.4% glucose, and appropriate antibiotics. At an OD_600_ = 2, the cultures were cooled to 18 °C and induced with 0.5 mM IPTG 1 h later. Expression continued overnight before the cells were harvested by centrifugation (10 min at 4500 × *g*). Lysis buffer (100 mM Tris, 800 mM NaCl, 10% glycerol, 10 mM imidazole, 0.5 mM TCEP, pH 8.0). Harvest of the cells was followed by sonication and affinity purification with a Ni-NTA column. The pooled fractions from the Ni-NTA column were either digested with TEV protease overnight (molar ratio of protein substrates vs. TEV-protease = 30:1) at 4 °C followed by purification of the cleaved protein by reverse IMAC or purified without His-tag cleavage via gel filtration on a Superdex 75 column. For the *A. pompejana* and *C. teleta* proteins used in crystallographic studies and DSF in Supplementary Fig. S2, an additional purification step on a HiTrap heparin HP column (GE Healthcare) was performed before the final gel filtration. Totally expressed, uncleaved and the cleaved purified protein was analyzed by mass spectrometry and SDS-PAGE (Supplementary Fig. S7).

### Protein crystallography and structure determination

Crystals of the *A. pompejana* DBD were grown at 20 °C with the sitting drop vapor diffusion technique using a Mosquito® crystallization robot (TTP Labtech) and SWISSCI 3-lens crystallization plates. Protein solution: 7.2 mg/ml in 25 mM HEPES, pH 7.5, 150 mM NaCl, 0.5 mM TCEP. Crystallization buffer: 22% PEG 3350, 0.2 M MgCl_2_, 0.1 M Tris, pH 8.3. Drop size: 300 nL (150 nL protein solution + 150 nL crystallization buffer). Crystals were cryoprotected with mother liquor supplemented with 23% ethylene glycol and flash-frozen in liquid nitrogen. X-ray data sets were collected at 100 K at beamline X06SA of the Swiss Light Source, Villigen, Switzerland. The diffraction data were integrated with XDS [70] and scaled with AIMLESS [71], which is part of the CCP4 program suite [72]. The structure was solved by molecular replacement with PHASER [73] using a homology model based on PDB entry 2XWR as a search model (generated using SWISS-MODEL [74]). The structure was then refined using iterative cycles of manual model building in COOT [75] and refinement in PHENIX [76]. A summary of the data collection and refinement statistics is given in Supplementary Table S1. Structural figures were prepared using PyMOL (www.pymol.org).

### Differential scanning calorimetry

Calorimetric measurements were made using a Microcal VP-Capillary-DSC (Malvern Instruments). Denaturation curves were obtained by heating up the p53 DBD (20 µM in PBS buffer, pH 7.0) from 10 to 85 °C, at a heating rate of 200 or 300 °C/h. As the denaturation of p53 family proteins was found to be irreversible, a final measurement of the denatured protein was made to obtain the baseline heat capacity without the higher-order structural transitions. Data were analyzed by using the Origin® software package provided with the DSC equipment. As the obtained curves could not be fitted to a two-state model, a non-two-state model settled to two peaks was used, and values obtained for the main species were considered for the records.

### Differential scanning fluorimetry (DSF)

In conventional DSF, melting temperatures of the p53 family DBDs were determined using the dye SYPRO Orange, which changes its fluorescence properties when binding to hydrophobic regions that become exposed upon thermal unfolding. Real-time melt analyses were performed using an Agilent MX3005P real-time qPCR instrument (excitation/emission filters = 492/610 nm). Proteins were assayed in a 96-well plate in a 25 mM HEPES, pH 7.5, 150 mM NaCl, 0.5 mM TCEP assay buffer with a final protein concentration of 2 μΜ and the fluorescent dye SYPRO Orange (Invitrogen) at a dilution of 1:1000 (total volume of 20 μL per well). The fluorescence signal was measured while increasing the temperature from 25 to 80 °C, at a heating rate of 180 °C/h. *T*_m_ values were calculated after fitting the fluorescence curves to the Boltzmann function. Measurements were performed in quintuplets.

For Prometheus nano DSF, thermal denaturation was performed on the Prometheus NT.48 (Nanotemper) from 20 to 95 °C (heating rate 300 °C/h) with 80% excitation laser power. The tryptophan fluorescence emission was monitored at 330 nm and 350 nm as a function of increasing temperature. 10 µl protein samples at a concentration of 0.3 mg/ml were filled into the capillaries, and the intrinsic fluorescence signal expressed by the 350 nm/330 nm emission ratio was plotted as a function of temperature. *T*_m_ values were determined using the peak of the first derivative of the melting curves.

### Circular dichroism

CD experiments were performed using an Aviv CD spectrometer model 215 equipped with a water-cooled Peltier unit. Thermal denaturation was followed by measuring the change in ellipticity (in millidegrees, mdeg) at 220 nm of 20 µM of protein in PBS buffer, pH 7.0, upon heating from 10 to 80 °C. Different heating rates were applied (60, 180, and 300 °C/h), and CD data were collected in increments of 1 °C. The experimental denaturation profiles were analyzed by mathematical treatment of sigmoidal curves using the Boltzmann fit function with Origin® software.

### Electrophoretic mobility shift assays

The Con1 DNA sequence for the EMSA was synthesized by Sigma Genosys (Israel) and purified with a reverse-phase cartridge. The sequence was designed as an intramolecular hairpin construct with 23 bp in the stem and five cytosines in the loop as previously described [58, 60].

Con1: cGGGCATGTCCGGGCATGTCCtg

For the EMSA shown in Fig. 7, protein samples with different concentrations (200–2400 nM) were incubated for 1 h with Con1 DNA at 4 or 20 °C and then run on a 6% polyacrylamide (37.5:1 acrylamide:bisacrylamide ratio) at the same temperature as the incubation until the bromophenol blue dye had migrated 8 cm. For the EMSA experiments in Supplementary Fig. S6 to monitor temperature dependence of DNA binding, protein samples (nominal concentration of 2400 nM) were incubated with Con1 DNA for 1 h at the indicated temperature, and then gels were run at 4 °C. Binding buffer composition: 200 mM NaCl, 10 mM MgCl_2_, 62.5 mM Tris-HCl (pH 7.5), 1 mM ATP, 12.5 mM DTT, 25 µg/ml BSA, 0.05% NP-40, 12.5% glycerol; the total ionic strength was 290 mM. The gels were run at 550 V in a running buffer containing 1× TG [25 mM Tris⋅HCl (pH 8.3), 190 mM glycine] until the bromophenol blue dye migrated 8 cm.

## Supplementary information

Supporting Information

reproducibility checklist

## Data Availability

The coordinates and structure factors of the *Alvinella pompejana* p53 homolog DBD were deposited in the Protein Data Bank under accession code 7PC6.
