# Supplementary material for: Evolutionary history of the p53 family DNA-binding domain: insights from an Alvinella pompejana homolog
Source: Cell Death Dis. 2022 Mar 7;13(3):214. doi: 10.1038/s41419-022-04653-8 (PMC8901663; doi:10.1038/s41419-022-04653-8)
Supplement: Supplementary file 1 — Supporting Information [file 41419_2022_4653_MOESM1_ESM.pdf]

Supporting Information

Evolutionary history of the p53 family DNA-binding domain: insights from an *Alvinella pompejana* homolog

Qiang Zhang[1], Dimitrios-Ilias Balourdas[2,3], Bruno Baron[4], Alon Senitzki[5], Tali E. Haran[*,5], Klas G. Wiman[*,6], Thierry Soussi[*,7,8,9] & Andreas C. Joerger[*,2,3,9]

[1] Department of Neuroscience, Biomedicum, Karolinska Institutet, Stockholm, Sweden

[2] Institute of Pharmaceutical Chemistry, Goethe University, Max-von-Laue-Str. 9, 60438 Frankfurt am Main, Germany

[3] Buchmann Institute for Molecular Life Sciences and Structural Genomics Consortium (SGC), Max-von-Laue-Str. 15, 60438 Frankfurt am Main, Germany

[4] Plateforme de Biophysique Moléculaire, Centre de Ressources et de Recherches Technologique (C2RT), Institut Pasteur, 75015 Paris, France

[5] Department of Biology, Technion-Israel Institute of Technology, Technion City, Haifa 32000, Israel

[6] Department of Oncology-Pathology, Bioclinicum, Karolinska Institutet, Stockholm, Sweden

[7]Department of Immunology, Genetics and Pathology, Uppsala University, Uppsala, Sweden

[8] Sorbonne Université, UPMC Univ Paris 06, 75005 Paris, France

*correspondence:

joerger@pharmchem.uni-frankfurt.de (A.C.J), thierry.soussi@sorbonne-universite.fr (T.S.), bitali@technion.ac.il (T.E.H.), or klas.wiman@ki.se (K.G.W.).

[9]These authors contributed equally

```
EST_N72937             1 SSSAPSPYPDNTQALSPSSLHNHLQSPAPTVPSNTPYSGEYGFEISFQHQSKET  54
GO114003.1             1 SSSAPSPYPDNTQALSPSSLHNHLQSPAPTVPSNTPYSGEYGFEISFQHQSKET  54
GO114002.1             1 ---------------HHHPSINHLQSPAXTVPSNTPYSGEYGXEISFQHQSKET  39
Protein crystallized   1 ---------------------------SMTVPSNTPYSGEYGFEISFQHQSKET  27

EST_N72937            55 KSTTWTFSESLKKLFVRMATTCPVRFKTVHQPPAGSVIRAMPIYVKPEHVQEVV 108
GO114003.1            55 KSTTWTFSESLKKLFVRMATTCPVRFKTVHQPPAGSVIRAMPIYVKPEHVQEVV 108
GO114002.1            40 KSTTWTFSESLKKLFVRMATTCPVRFKTVHQPPAGSVIRAMPIYVKPEHVQEVV  93
Protein crystallized  28 KSTTWTFSESLKKLFVRMATTCPVRFKTVHQPPAGSVIRAMPIYVKPEHVQEVV  81

EST_N72937           109 KRCPNHATTKEHNEDHPAPTHLVRCEHKLASYVEDPYTGRQSVIIPQEHPQAGA 162
GO114003.1           109 KRCPNHATTKEHNEDHPAPTHLVRCEHKLASYVEDPYTGRQSVIIPQEHPQAGA 162
GO114002.1            94 KRCPNHATTKEHNEDHPAPTHLVRCEHKLASYVEDPYTGRQSVIIPQEHPQAGA 147
Protein crystallized  82 KRCPNHATTKEHNEDHPAPTHLVRCEHKLASYVEDPYTGRQSVIIPQEHPQAGA 135

EST_N72937           163 EWVTNLYQFMCFSSCVGGLNRRPIQVIFTLEHEGVVLGRQAVEVRICACPGRDR 216
GO114003.1           163 EWVTNLYQFMCFSSCVGGLNRRPIQVIFTLEHEGVVLGRQAVEVRICACPGRDR 216
GO114002.1           148 EWVTNLYQFMCFSSCVGGLNRRPIQVIFTLEHEGVVLGRQAVEVRICACPGRDR 201
Protein crystallized 136 EWVTNLYQFMCFSSCVGGLNRRPIQVIFTLEHEGVVLGRQAVEVRICACPGRDR 189

EST_N72937           217 RAEETAADPNKQQKRPANKMTISTEMTSVGPALKP                    251
GO114003.1           217 RAEETAADPNKQ-----------------------                    228
GO114002.1           202 RAEETAADPNKQQKRPANKMTISTEMTSVGPALKP                    236
Protein crystallized 190 RAEETAADPNKQQKR--------------------                    204
```

Figure S1. Translation of *Alvinella pompejana* p53 homolog ESTs and sequence of the protein construct used for X-ray crystallography.

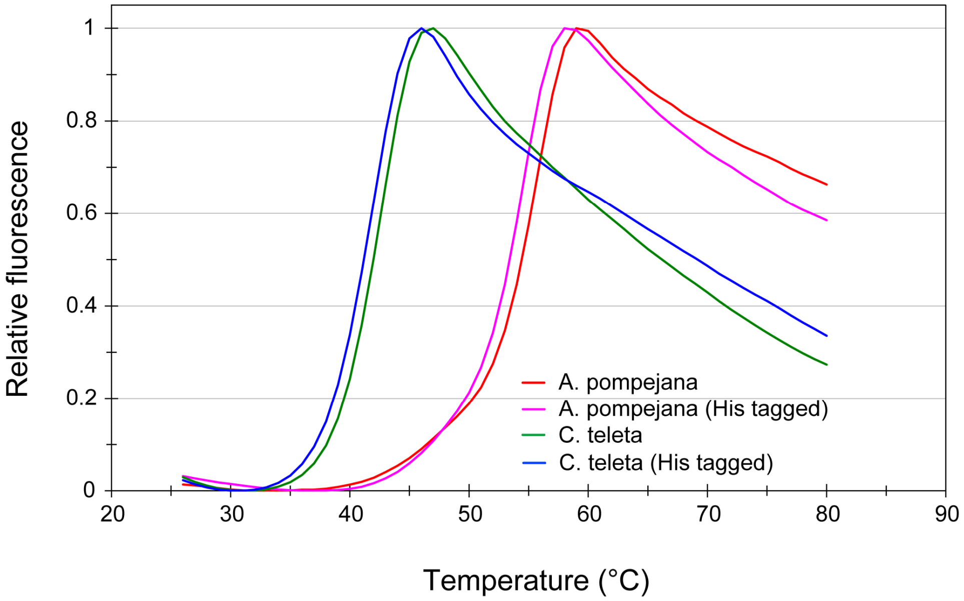

Figure S2. Thermal unfolding of annelid worm p53 family DBDs monitored by DSF with SYPRO orange. Normalized raw data of DSF analysis at a heating rate of 180 °C/h for the DBDs of *Alvinella pompejana* and *Capitella teleta* (with and without N-terminal hexahistidine tag) showing increased thermostability of the *Alvinella pompejana* DBD. In both cases, the presence of an N-terminal hexahistidine tag had a minor effect on DBD stability, lowering the T_m by about 1 °C.

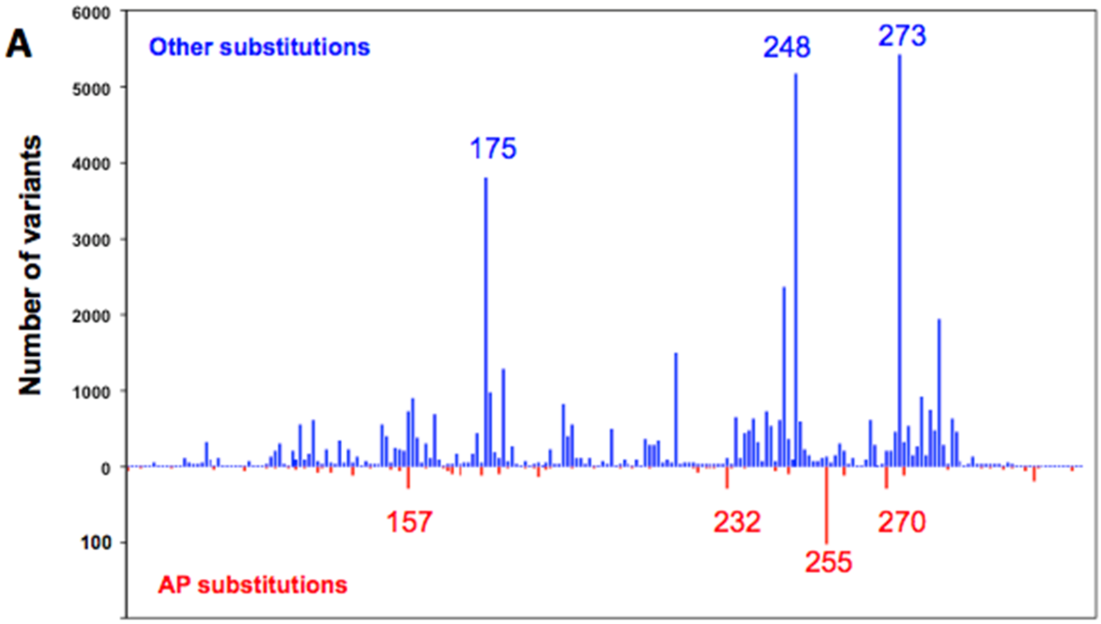

B

Variants	AP substitutions	Other substitutions
p.Q144R	12	54
p.V157I	30	718
p.Q167E	10	24
p.M169V	13	32
p.R174K	13	61
p.H178N	10	110
p.G187D	15	56
p.I232N	29	109
p.M246L	10	357
p.I255F	103	128
p.D259H	12	204
p.F270V	29	205
p.V274I	12	319
p.P309S	20	8

Figure S3. Distribution of p53 cancer variants in the DNA-binding domain of the human p53 protein. (A) For each position in the protein (p53 residues 100-300, ref NP_000537.3) the number of variations is shown. AP substitutions (red, lower part): number of p53 cancer variants leading to an amino acid substitution corresponding to the wild-type residue in the *Alvinella pompejana* protein. Other substitutions (blue, upper part): number of p53 variants leading to an amino acid substitution not identical to the wild-type residue in the *A. pompejana* protein. (B) Table showing the most frequent p53 variants identified in the above graph. The frequency of the different cancer mutations is based on the 2017 issue of the TP53 database (http://p53.fr, 80,000 variants) [1].

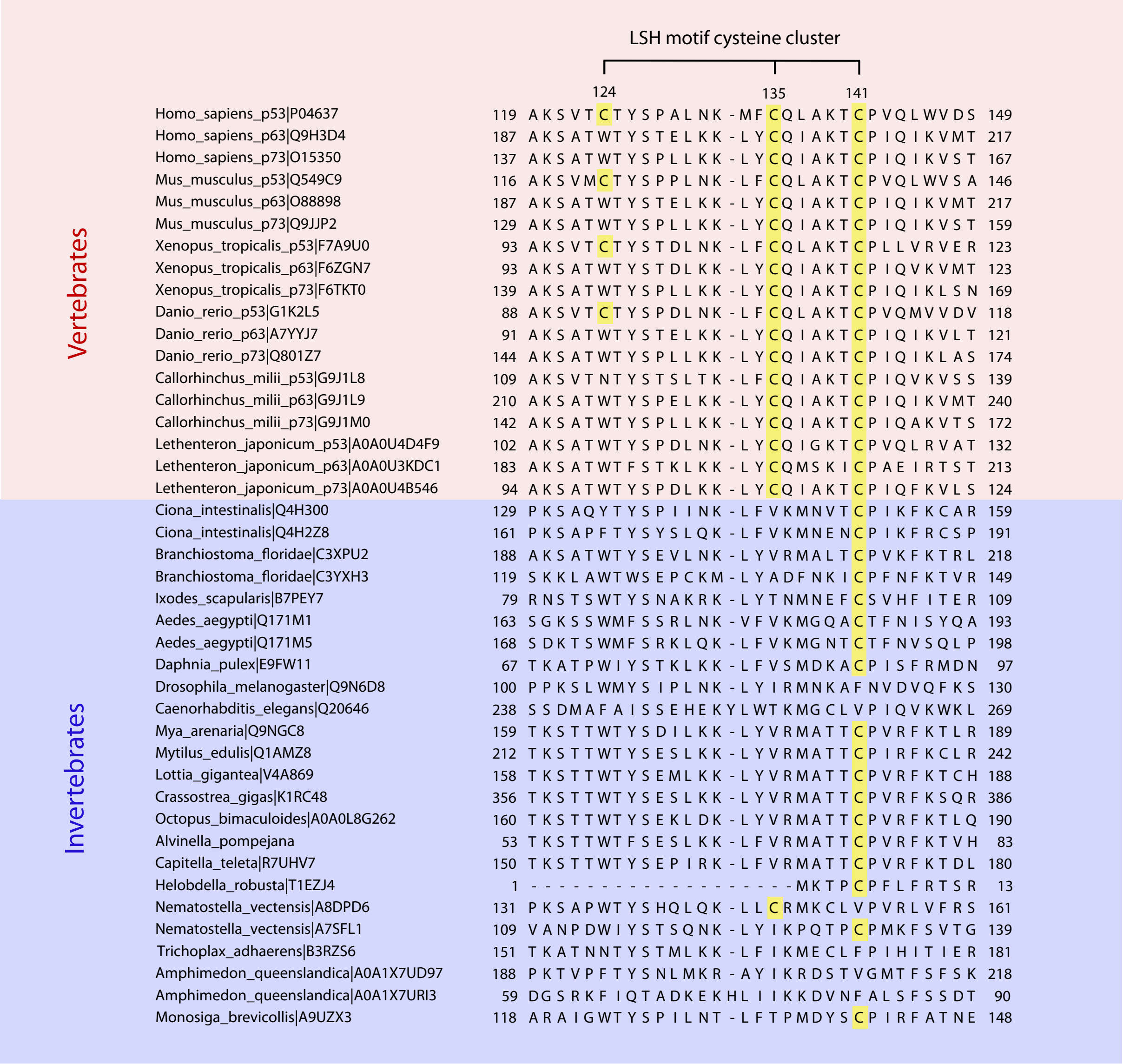

Figure S4. Conservation of the three-cysteine cluster in the loop-sheet-helix motif in the DNA-binding domain of p53 family proteins. Sequences were aligned using Jalview [2] with manual editing based on known crystal structures. UniProt accession codes are given after the species and name of the protein. This alignment suggests a gradual appearance of the three cysteines during p53 evolution, with Cys141 (human p53 numbering) possibly already present in the last common ancestor of all extant animals, Cys135 appearing shortly before the radiation of vertebrates and Cys124 last, as a vertebrate p53 specific variation, first observed in bony fishes.

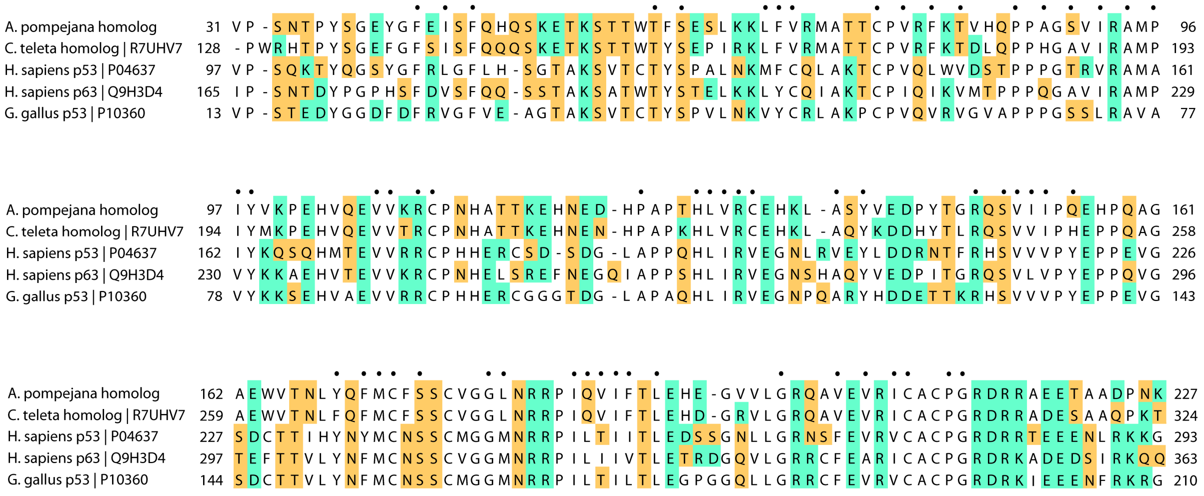

Figure S5. Sequence alignment and CvP-bias of p53 family DBDs. Sequences were aligned using Jalview [2]. Accession codes are given after the species and the name of the protein. CvP-bias = ($N_{charged}$ - N_{polar})/length x 100. $N_{charged}$ = number of charged amino acids (E, D, K, R; highlighted in green), N_{polar} = number of polar amino acids (S, T, N, Q; highlighted in orange). CvP values were calculated using the domain boundaries shown in the alignment. Higher CvP values have been associated with higher thermostability of orthologs [3]. This correlation is not seen when comparing the p53 family DBDs of the thermophilic *A. pompejana* and the mesophilic *C. teleta* annelids, which have CvP values of 0.5 and 1.5, respectively. These values were slightly higher than for the human p63 DBD (-1.5) and the human p53 DBD (-1.0). The CvP value of the chicken (*G. gallus*) p53 DBD was much higher (9.1), supporting the notion that the high stability of chicken p53 compared with the human homolog is, at least in part, due to the higher number of charged surface residues [4]. Buried side chains are marked with a dot. Unmarked residues have side chains with a solvent accessibility of ≥10% in the crystal structure of the human p53 DBD (PDB entry 2XWR, chain A) as calculated with the software NACCESS, which uses a probe radius of 1.4 Å for calculating solvent-accessible areas.

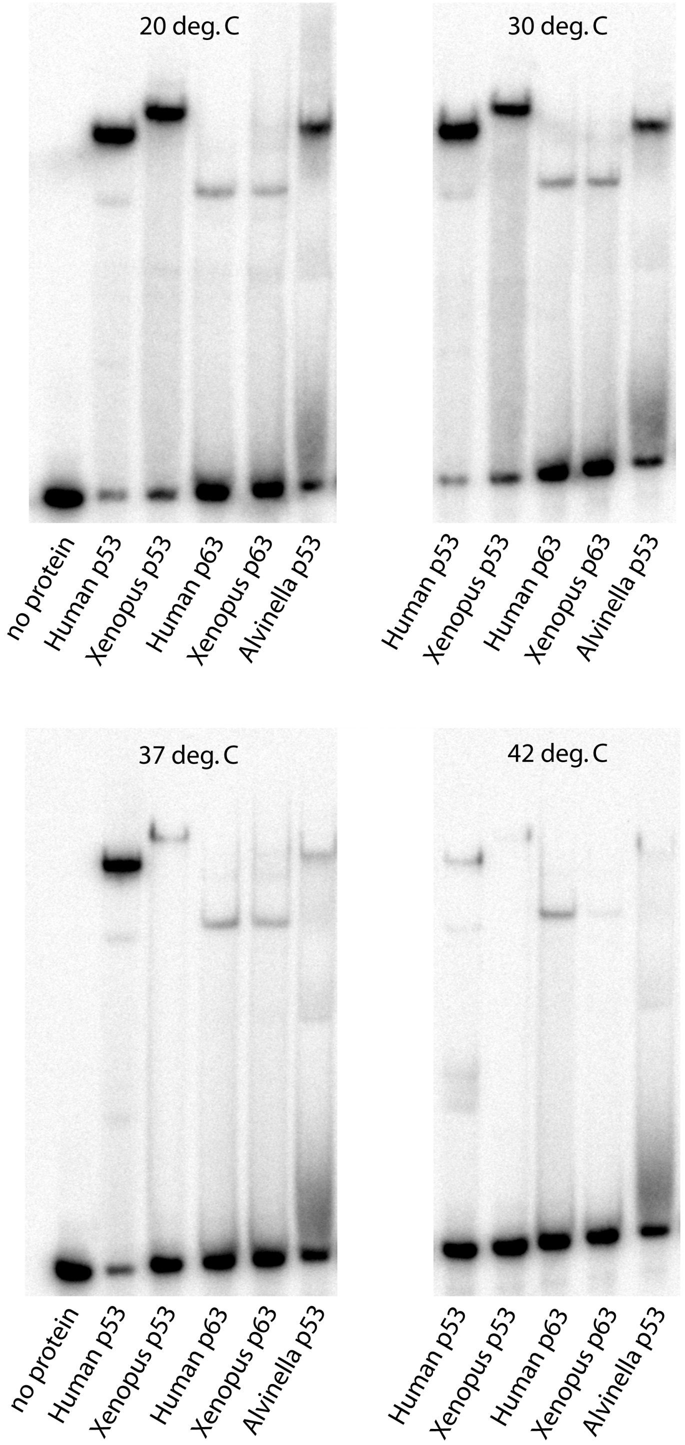

Figure S6. EMSA of p53 family DBDs binding to Con1 DNA at various temperatures. DBDs (nominal concentration of 2400 nM) were incubated with Con1 DNA for 60 min at the indicated temperature and then run on a 6% polyacrylamide gel (37.5:1 (acrylamide: bisacrylamide ratio) at 4 °C.

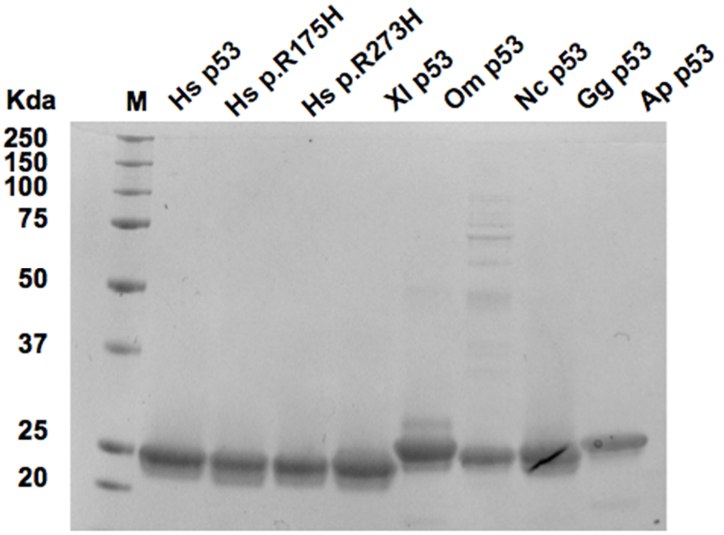

Figure S7. Polyacrylamide gel electrophoresis of representative DBD samples used in the present study. The following abbreviations are used: Hs p53, human p53; Hs p.R175H, human p53 hot spot mutant R175H (structural mutant); Hs p.R273H, human p53 hot spot mutant R273H (DNA-contact mutant); Xl p53: *Xenopus laevis* p53; Om p53, *Oncorhynchus mykiss* (rainbow trout) p53; Nc p53, *Notothenia coriiceps* (Antarctic fish) p53 (not used in the present study); Gg p53, *Gallus gallus* (chicken) p53; Ap p53, *Alvinella pompejana* p53 homolog.

Table S1. X-ray data collection and refinement statistics

	Alvinella pompejana p53 homolog DNA-binding domain
Data Collection	
Space Group	*P*1
a, b, c (Å),	55.90, 69.99, 114.85
α, β, γ (°)	88.07, 89.92, 81.51
Molecules/asymmetric unit	8
Resolution (Å)[a]	49.7-1.92 (1.95-1.92)
Unique reflections	121,535
Completeness (%)[a]	92.5 (93.0)
Multiplicity[a]	3.7 (3.8)
R_{merge} (%)[a]	5.6 (83.5)
CC(1/2)[a]	0.998 (0.630)
Mean $I/\sigma(I)$[a]	12.5 (1.7)
Refinement	
R_{work}, (%)[b]	18.4
R_{free}, (%)[b]	22.5
No. of atoms	
Protein[c]	11,898
Zinc	8
Water	860
Ethylene glycol	16
RMSD bonds (Å)	0.007
RMSD angles (°)	0.83
Mean *B* (Å^2)	38.2
Ramachandran favored (%)[d]	97.7
Ramachandran outliers (%)[d]	0.1
PDB entry	7PC6

[a]Values in parentheses are for the highest-resolution shell.

[b]R_{work} and $R_{free} = \sum||F_{obs}| - |F_{calc}||/\sum|F_{obs}|$, where R_{free} was calculated with 5 % of the reflections chosen at random and not used in the refinement.

[c]Number includes alternative conformations.

[d]MolProbity [5] statistics

SI references

1. Leroy, B. et al. The TP53 website: an integrative resource centre for the TP53 mutation database and TP53 mutant analysis. *Nucleic Acids Res* **41**, D962-9 (2013).
2. Waterhouse, A.M., Procter, J.B., Martin, D.M., Clamp, M. & Barton, G.J. Jalview Version 2--a multiple sequence alignment editor and analysis workbench. *Bioinformatics* **25**, 1189-91 (2009).
3. Holder, T. et al. Deep transcriptome-sequencing and proteome analysis of the hydrothermal vent annelid Alvinella pompejana identifies the CvP-bias as a robust measure of eukaryotic thermostability. *Biol Direct* **8**, 2 (2013).
4. Khoo, K.H., Andreeva, A. & Fersht, A.R. Adaptive evolution of p53 thermodynamic stability. *J Mol Biol* **393**, 161-75 (2009).
5. Williams, C.J. et al. MolProbity: More and better reference data for improved all-atom structure validation. *Protein Sci* **27**, 293-315 (2018).
